# Recovery of Alumina Nanocapacitors after High Voltage Breakdown

**DOI:** 10.1038/s41598-017-01007-9

**Published:** 2017-04-20

**Authors:** A. Belkin, A. Bezryadin, L. Hendren, A. Hubler

**Affiliations:** Department of Physics, University of Illinois at Urbana-Champaign, 1110 W. Green str., Urbana, IL 61801 USA

## Abstract

Breakdown of a dielectric material at high electric fields significantly limits the applicability of metal-dielectric-metal capacitors for energy storage applications. Here we demonstrate that the insulating properties of atomic-layer-deposited Al_2_O_3_ thin films in Al/Al_2_O_3_/Al trilayers can recover after the breakdown. The recovery has been observed in samples with the dielectric thickness spanning from 4 to 9 nm. This phenomenon holds promise for a new generation of capacitors capable of restoring their properties after the dielectric breakdown. Also, if employed in capacitor banks, the recovery process will ensure that the bank remains operational even if a breakdown occurs.

## Introduction

Recently, it has been suggested that the energy density in metal-dielectric-metal (MDM) capacitors could be significantly increased if the gap spacing between the electrodes is reduced to a nanometer scale^1, 2^. The improvement is expected because in vacuum capacitors the field enhancement factor, *β*, characterizing the local enhancement of the average electric field due to surface defects, decreases with the gap^1, 3, 4^. Hence, capacitors with the thickness of the insulating layer on the order of few nanometers, called nanocapacitors, might be able to withstand much higher electric fields before a breakdown damaging the dielectric takes place.

In capacitors, where two electrodes are separated by a vacuum gap, breakdown discharges do not necessarily lead to a permanent damage of the capacitor plates. The initial breakdown could increase the field at which the subsequent breakdown is observed^4–6^. This phenomenon, called the breakdown conditioning, is attributed to the destruction of the surface defects such as protrusions, which are usually responsible for the breakdown. Similarly, in capacitors with certain dielectrics^7–10^ and thin metal electrodes breakdowns do not unavoidably cause shorts. On breakdown the metal could melt or evaporate locally around the breakdown hole, insulating it from the rest of the capacitor. These types of dielectric failure are usually called “self-healing” breakdowns.

In gas circuit breakers, widely used in high-voltage transmission and distribution networks, the dielectric breakdown plays the key role for a successful interruption of high short circuit currents^11, 12^. The subsequent recovery of a gaseous dielectric, during which the gas recombines, cools, and recovers its initial dielectric strength, is one of the main stages of the breaker operation. Due to the complexity of the involved physical processes an understanding of the dielectric recovery continues to be an area of active research.

There are different models regarding the breakdown mechanism in dielectrics^13, 14^, including hole-induced breakdown model^15–18^, the electron-trapping breakdown model^19^, resonant-tunneling-induced breakdown model^20^ and filamentary model^21^. Despite all the debates, it is generally accepted that dielectric failure can be described as the two-step process, *i*.*e*. wearout^22^ followed by the breakdown^23^. During the wearout phase, the defects are generated inside^19^ or at the interface^24^ of the dielectric by the applied electric field or by the leakage currents flowing through the dielectric. Ultimately the density of these defects becomes sufficient to lead to locally high current densities followed by thermal runaway in a confined region^25^. The time it takes to accumulate the critical amount of defects, *i*.*e*. mechanism of the time-dependent dielectric breakdown, depends on various factors. Among them are the strength of the electric field^26^ and the type of the charge carrier transfer (Fowler-Nordheim, Frenkel-Poole or direct tunneling)^27^, which in turn depends on the dielectric material and its thickness.

If the time required to recover the capacitor after breakdown is shorter than the time to dielectric breakdown of other capacitors in capacitor banks^28, 29^, then it should be possible to avoid using distribution automation/power management systems^30^ to prevent power outage.

In this work, we investigate a dielectric breakdown in Al/Al_2_O_3_/Al nanocapacitors. We observe two types of breakdown: local and global. Local breakdown appears as a nanometer size crater visible under a scanning electron microscope. It does not produce any noticeable signature on the voltage-current characteristic. Thus, we assume it is a self-healing phenomenon. We also observe a global breakdown that appears as a current jump on the voltage-current curve. We find that this type of the breakdown also shows a recovery effect: when the voltage is reduced the conductance decreases again. Both of these recovery and self-healing phenomena are of significant importance since they can help to improve the reliability of capacitor-based energy storage systems. We also find that these effects of recovery and self-healing are the most pronounced in nanocapacitors, *i*.*e*. capacitors where an insulating layer is just a few nanometers thick. Thus, our finding indicates that nanocapacitors are promising for the creation of powerful energy storage systems.

## Sample Description and Fabrication

**Figure 1:**
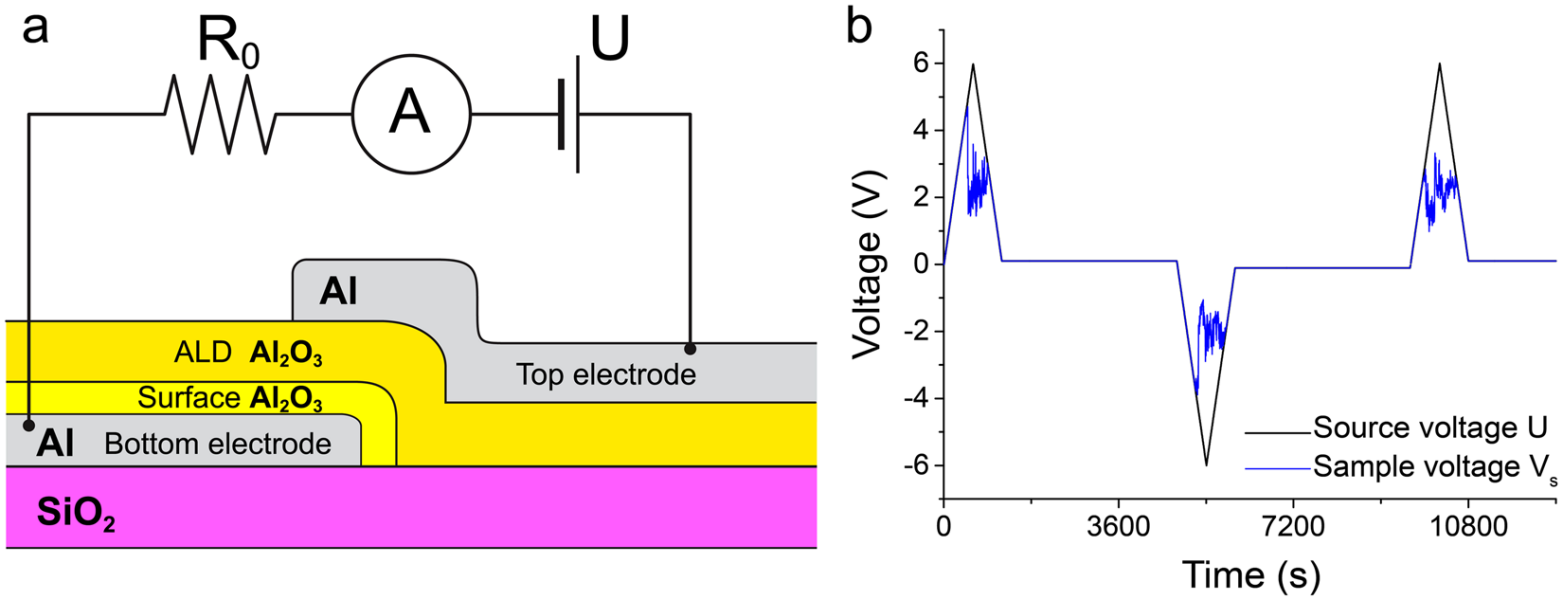
(**a**) Schematic of the sample (not to scale) and electrical diagram of the measurement setup. All the layers are labeled explicitly. The measurement setup includes electrometer Keithley 6517B serving as the voltage source and the ammeter, and the series resistor R_0_ ≈ 10 MΩ. (**b**) An example of an electric breakdown measurement. Black line shows how the source voltage changes over time, the blue line depicts the corresponding voltage drop on the sample. The thickness of the insulating layer in this example was 4 nm. The sample voltage at first follows the source voltage. In this example, at voltage about 5 V dielectric breakdown happens. The sample resistance gets reduced by orders of magnitude, thus the voltage on the capacitor (blue curve) also drops.

The studied MDM capacitors, which schematic is shown in Fig. 1(a), were grown on highly resistive <100> Si wafer capped by 280 nm SiO_2_ film (Silicon Quest Int’l, Si resistivity is 10^4^ Ohm · cm at 300 K). The substrates were successively sonicated for 3 minutes in acetone, isopropanol, deionized water, nitric acid, and then again in deionized water and isopropanol. The bottom aluminum electrode was patterned on the substrate by means of photolithography followed by an Al deposition and a lift-off procedure. It has a square shape with the edge length equal to 100 μm. The deposition of Al was done in the electron-beam evaporator with the base pressure ~1 × 10^−9^ Torr. The thickness of the bottom electrode was 30 nm (deposition rate ~1 Å/s). Before removing the sample from the evaporator, it was oxidized in the pure oxygen atmosphere (partial pressure ~3 Torr) during 1 hour in order to create a high-quality surface oxide using a controlled pure oxygen environment. As a result, an amorphous^31, 32^ oxide film with the thickness about 15 Angstroms was formed on the aluminum surface^33^.

Subsequently, the sample was transferred into an atomic layer deposition (ALD) system. There, the entire surface of the sample was coated with a few nanometer thick layer of aluminum oxide. The desired thickness of the dielectric layer was deposited at 80 °C using tri-methyl aluminum and water. While the samples were moved from the Al deposition chamber to the ALD chamber, they were exposed to air for a short period of time (on the order of 1 hour). As it is known, Al oxidation rate drastically slows down after the alumina thickens to about 15 Angstroms^33^. The thickness of 20 Angstroms can be reached after ~25 days. A similar behavior is observed when the film oxidizes in air at room temperature^34^. We expect that the increase of the dielectric thickness on the bottom electrode due to the air exposure was negligible, thus the thickness of naturally-grown oxide layer did not exceed 1.5 nm. The main part of the insulating film was then deposited in the ALD setup.

The final step, *i*.*e*. the fabrication of the top Al electrode, was done by using a metal shadow mask that rendered the rectangular 1 × 3 mm^2^ stripe overlaying the bottom square electrode. Its thickness was 70 nm, which was enough to form a continuous film partially covering the bottom electrode (previously coated with the insulating aluminum oxide layer) and the substrate (see Fig. 1(a)).

## Measurement Details

The source of the dc bias voltage, *U*, was connected in series with resistor, *R*_*0*_, sample and ammeter, *A* (see Fig. 1(a)). The source voltage *U* increased and then decreased at the constant rate equal to 0.01 V/s (see the example in Fig. 1(b)). The amplitude of the voltage was fixed for each set of samples with a particular dielectric thickness. We added one-hour delays between positive and negative voltage ridges, where it stayed at near-zero level (a small bias of 0.1 V or −0.1 V was applied to monitor the evolution of the sample resistance). The resistance of the capacitor is defined as *R*_*s*_ = *U*/*I* − *R*_*0*_, where *I* is the current in the circuit. Before the breakdown event, the resistance of the sample exceeds *R*_*0*_ by several orders of magnitude. Hence, a drop of the potential on the sample, *V*_*s*_, and the source voltage *U* are almost identical. This is confirmed by Fig. 1(b) where the black and blue curves, representing *U* and *V*_*s*_, at first follow each other. As *U* is ramped up, *V*_*s*_ reaches a breakdown value, *V*_*b*_, specific for each sample. At that moment the current in the circuit jumps up to a value limited by the series resistor, *i*.*e*. *V*_*b*_/*R*_*0*_ (the current can be less than this if the sample resistance is still high or comparable to *R*_*0*_). As a result, *V*_*s*_ = *U* − *IR*_*0*_ abruptly goes down and deviates from *U* (see Fig. 1(b)).

## Results

**Figure 2:**
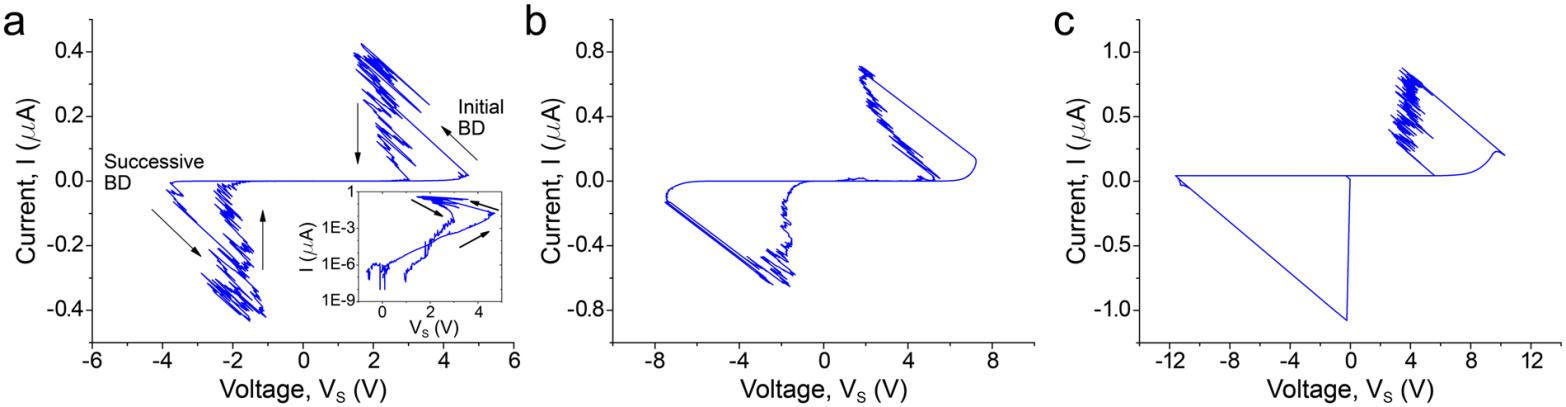
Current-voltage dependences of samples with alumina thicknesses equal to (**a**) 4 nm, (**b**) 6.5 nm and (**c**) 9 nm. The data plotted here corresponds to the first full period of the voltage curve (see Fig. 1(b)). Source voltage amplitude is (**a**) 6 V, (**b**) 9 V and (**c**) 12 V. Arrows serve as a guide to an eye. V_s_ is the voltage between capacitor plates.

Typical examples of the current-voltage characteristics of the samples with dielectric thicknesses 4, 6.5 and 9 nm are shown in Fig. 2. For the increasing source voltage *U*, the sample voltage *V*_*s*_ and the leakage current *I* continue to increase (inset in Fig. 2(a)) until the initial breakdown occurs (marked “Initial BD” in Fig. 2(a)). After that event the sample current jumps up but the sample voltage falls down despite an ongoing growth of the source voltage. Thus, we observe a bend in *I*-*V* curves towards lower-*V*_*s*_/higher-*I* region. During the reverse passage, *i*.*e*. when the source voltage is ramped down after the breakdown, the current through the sample changes such that the product *IR*_*0*_ decreases faster than voltage *U*, leading to the shift of the *I*-*V* curve towards higher-V_s_/lower-*I* region (see Fig. 2). Interestingly, each *I*-*V* curve shown in Fig. 2 has the second distinct jump at negative bias voltage (marked “Successive BD” in Fig. 2(a)). Since the electric field magnitudes for positive and negative breakdowns are comparable (*E* ≈ 1.2 ÷ 1.3 V/nm) and the existence of the successive breakdowns was verified for ~20 measured samples, we conclude that the studied nanocapacitors can survive the dielectric breakdown (otherwise there would be just one breakdown event), which is the key result of the present work. Let us note that samples with thinner dielectric, *i*.*e*. 4 and 6.5 nm, exhibit a gradual increase of the current prior to a breakdown. At the same time, in thicker samples with 9 nm of alumina a growth of the current is replaced by its decrease before a jump takes place (see Fig. 2(c)). Such a change corresponds to a negative differential resistance of the studied nanocapacitors, which has been previously observed in different MDM systems (see e.g. refs 35 and 36 for review).

**Figure 3:**
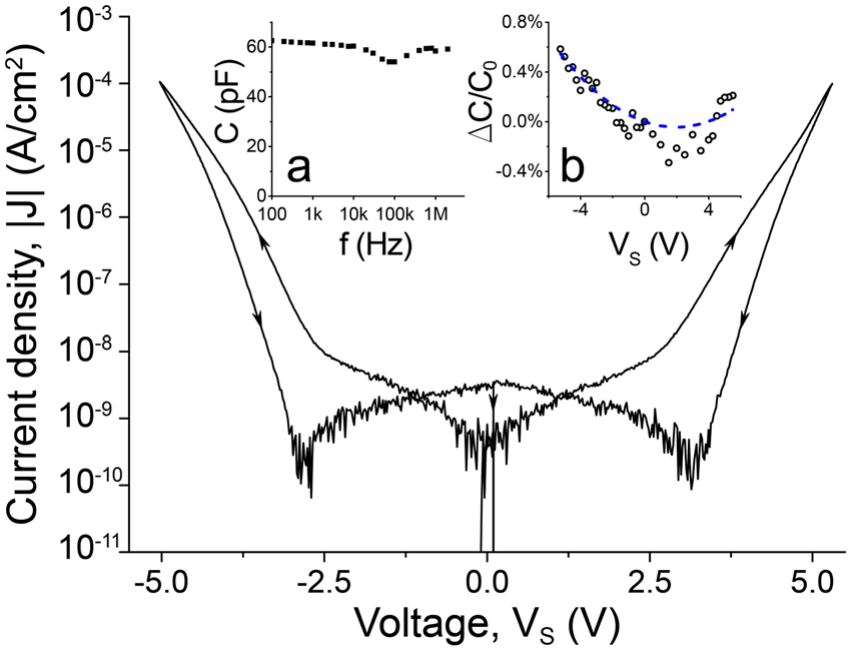
The dependence of the absolute value of the leakage current density, |J|, on the sample voltage, V_S_, for the nanocapacitor with 9 nm Al_2_O_3_ layer. Black arrows indicate the sections of the dependence corresponding to forward and backward voltage ramp. Each half cycle lasts 1800 s. Inset (**a**) is the dependence of the capacitance on the frequency of the voltage signal with the amplitude equal 1 V. Inset (**b**) shows the dependence of the normalized capacitance ΔC/C_0_ = (C − C_0_)/C_0_ on the applied constant bias voltage (black circles). To exclude the hysteresis from the analysis, we use data points obtained during the voltage sweep from +5 V to −5 V. Blue dashed line corresponds to the parabolic fit of the data.

Prior to reaching the breakdown, we investigated, on some samples, *I*-*V* dependences in the lower voltage region. An example of such measurements is given in Fig. 3. The observed hysteretic behavior reflects the influence of charge trapping in the dielectric^37^. Indeed, once the voltage is ramped up charges are pushed into the dielectric. However, not all charges reach the opposite electrode. Some of them get trapped in various defects inside the insulating film. As the electric field is reversed, these trapped charges start to escape from the traps and move in the opposite direction. Such trap neutralization shifts the total sample current to lower values (see Fig. 3). At *V*_*s*_ ≈ 3 V (for 9 nm alumina sample) we observe the reversal of the current flow direction, which appears as a cusp on the absolute current versus voltage graph^38^. In addition, the capacitance-frequency and capacitance-voltage properties of the capacitors have been tested using QuadTech 7600 Plus LCR meter. The former type of measurements has revealed that the capacitance of the studied samples is nearly independent on the voltage signal frequency in the range from 100 Hz to 2 MHz (see inset (a) in Fig. 3). The presented data has been obtained by subtracting from the capacitance of the sample measured with thin-film leads connected to the MDM capacitor the capacitance of the sample with one of the leads cut. The origin of a small dip observed near 100 kHz is currently unclear. The voltage measurements, on the other hand, do show a capacitance change with the change of the applied voltage (see inset (b) in Fig. 3). The amplitude and frequency of the ac test voltage signal were 100 mV and 20 kHz, correspondingly. By fitting the data (dashed blue line in Fig. 3(b)) with a second order polynomial function *C*(*V*) = *C*_*0*_(*V*)(*αV* ^*2*^ + *βV* + 1), where *C*_*0*_(*V*) is the capacitance at zero bias, we obtain the following quadratic and linear voltage coefficients^38^ of the capacitance: *α* = 113 ppm/V^2^, *β* = −446 ppm/V.

**Figure 4:**
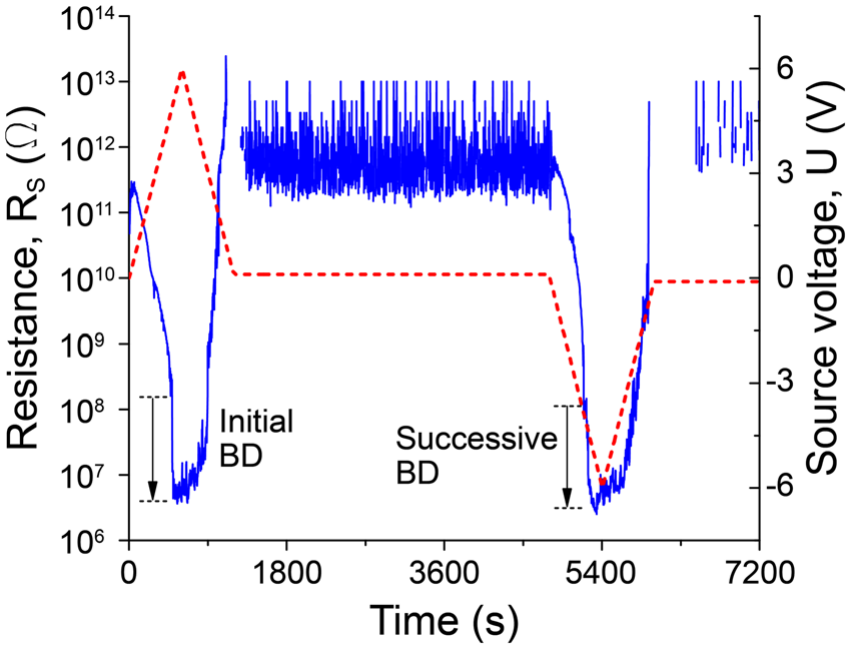
The time dependence of the source voltage U (red dashed line) and sample resistance R_s_ (blue line) for the nanocapacitor with 4 nm Al_2_O_3_ layer. Initial and successive global breakdown jumps are marked with black arrows.

The recovery process in Al/Al_2_O_3_/Al nanocapacitors can be demonstrated by plotting how the applied voltage, *U*, and the sample resistance, *R*_*s*_, change with the time. Let us make a brief analysis for 4 nm-thick sample, whose voltage-current characteristic is shown in Fig. 2(a). While the applied voltage is ramped up from zero, the current through the dielectric grows nonlinearly, thus the sample resistance decreases from approximately 100 GΩ to 100 MΩ (see Fig. 4). When the voltage reaches ~5 V, the initial global breakdown takes place. After the jump the resistance of the capacitor becomes rather low, that is about 5 MΩ. Such low resistance makes the capacitor basically nonfunctional. As the voltage is reduced a recovery process is observed. Namely, by the time the voltage drops to ~3 V the resistance increases by orders of magnitude and as the voltage approaches zero the resistance climbs as high as ~1 TΩ (see Fig. 4). During the delay time between voltage ridges with opposite polarities *R*_*s*_ stays near that 1 TΩ level.

Qualitative character of the resistance curve does not change as the voltage is varied in the negative region: *R*_*s*_ decreases from ~1 TΩ to ~100 MΩ, then the successive global breakdown jump occurs and as *U* approaches 0 V the resistance goes back up to 1 TΩ range. Note that the apparent discontinuity of the resistance curve on the logarithmic scale, such as observed in Fig. 4 in the ranges from 1100 s to 1300 s and from 6000 s to 6500 s, is due to the process of trap neutralization, which causes the charge to flow in the direction opposite to the applied field and thus effectively creates a “negative resistance”.

**Figure 5:**
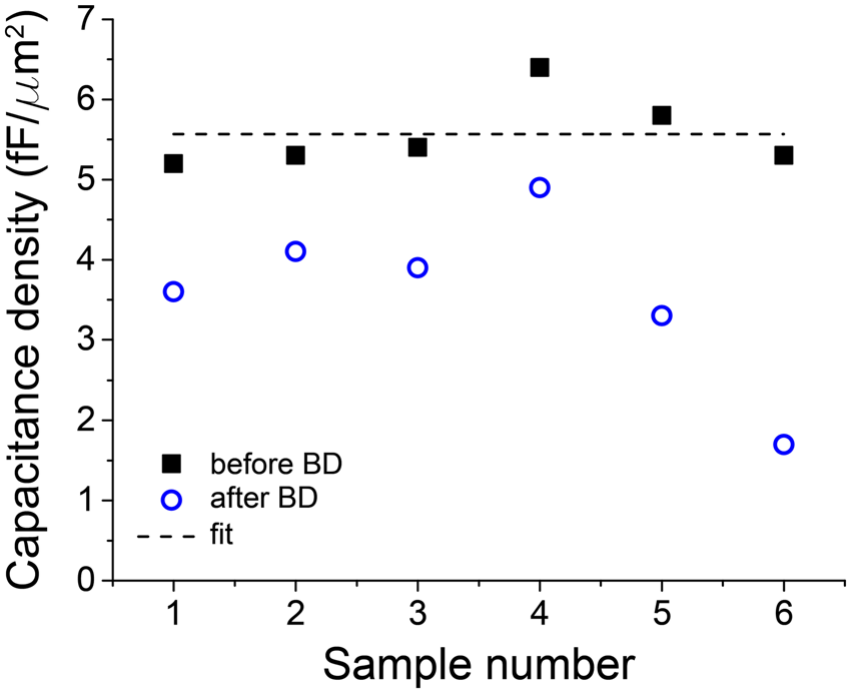
The capacitance of the samples before (black squares) and after (blue circles) the initial global breakdown. The thickness of the dielectric is 6.5 nm. Dashed line depicts the average value of the capacitance obtained before breakdown.

To investigate the efficiency of the recovery process further, we have tested the capacitance of the sample before the global breakdown (“Before BD”) and after the global breakdown (“After BD”). The capacitance was measured at fixed frequency and fixed amplitude of the test signal equal to 1 kHz and 0.7 V, correspondingly. Dielectric constant extracted from the averaged experimental value (dashed line in Fig. 5) of the data collected before the breakdown on six samples (black squares in Fig. 5) is ϵ_fit_ = 4.1 ± 0.2. This value matched well the dielectric constant of an ALD-grown thin Al_2_O_3_ film, ϵ ≈ 4.5, previously reported by Groner^39^. The recovery process appears rather efficient since a capacitance measured after the breakdown (blue circles in Fig. 5) is usually close to an initial one. The observed modest reduction of the capacitance is due to the occurrence of nanoscale “craters” near the points on the capacitor where the breakdown takes place. These craters, discussed below, isolate the damaged region from the good part of the capacitor plate.

## Discussion

**Figure 6:**
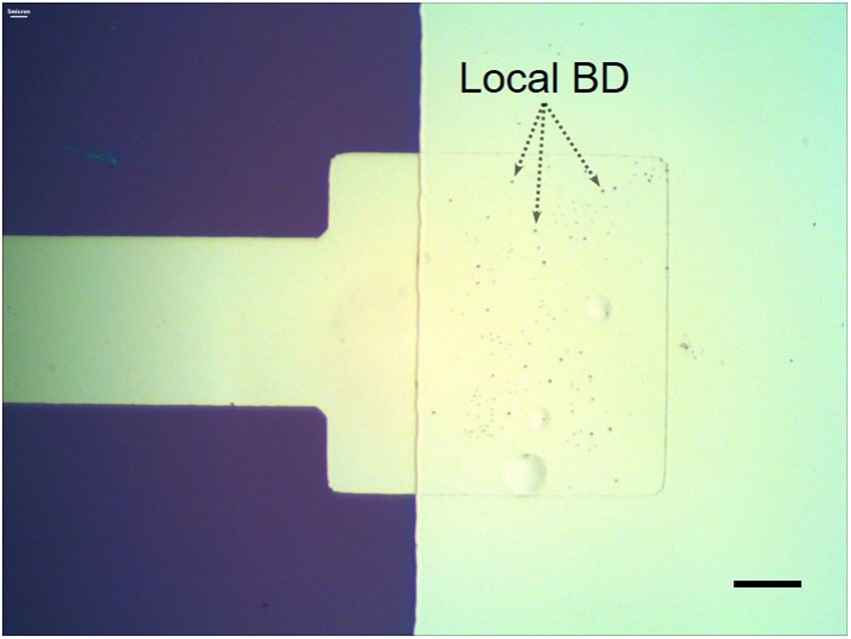
Optical image of a test MDM nanocapacitor after a high-voltage breakdown. Small black specks on the surface of the top electrode constitute local breakdowns (marked as “Local BD”). Scale bar corresponds to 20 μm.

**Figure 7:**
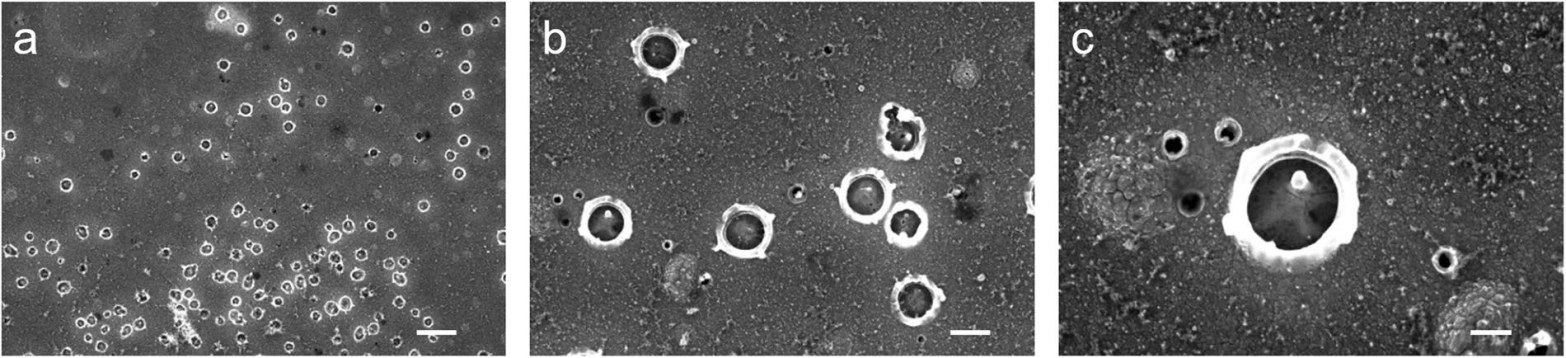
Scanning electron microscope images of the top surface of a test MDM nanocapacitor after a high-voltage breakdown made at (**a**) 5 k magnification, scale bar is 2 μm, (**b**) 20 k magnification, scale bar is 500 nm, and (**c**) 50 k magnification, scale bar is 200 nm.

The results presented above clearly show that a recovery process is possible in Al/Al_2_O_3_/Al nanocapacitors. A naive understanding of this process would be to say that some sort of tunneling of the electrons takes place through the insulating barrier of the MDM capacitor, and since the electric field is high, of the order of 1 V/nm, the tunneling becomes much stronger at high voltage due to the related barrier suppression. Yet, such simple picture does not explain the jump-wise increase of the current at a critical voltage and the existence of successive breakdowns observed in most of the experiments, the example being Fig. 2. In order to qualitatively explain the breakdown and the recovery phenomenon let us add a few additional observations. First, we find, by means of observations under an optical microscope, that above certain electric field (~1 V/nm) black specks start to emerge on the surface of the top electrode (see Fig. 6). The number of these specks rapidly increases with the voltage, but their appearance does not immediately lead to a breakdown jump. Interestingly, voltage-current dependences in our measurements do not have any peculiarities in the region where the specks develop. The nature of the observed phenomenon appears similar to that observed by Klein *et al*.^9^ in SiO_2_ films. Namely, a single speck is due to the discharge of the capacitor into a defect in the dielectric. The discharge is produced when the current through the defect causes a thermal instability and an increase in conductance by several orders of magnitude. The Joule heat causes evaporation and eruption at high mechanical pressure, and formation of the crater-like defect, the detailed structure of which is presented in Fig. 7. Since the metal evaporates around such breakdown spot, it does not short capacitor’s electrodes^9^. The above phenomenon has been previously referred to as the self-healing breakdown^7–10, 40^. The timescale of the breakdown event, τ_B_, can be estimated from the energy balance equation *E*_*m*_ = *E*_*J*_, where *E*_*m*_ is the energy required to melt the material inside a crater, and *E*_*J*_ is the Joule heating generated during the breakdown. *E*_*m*_ is proportional to the specific heat of the alumina (~900 J/kg K), the local temperature change (~2000 K) and the alumina mass confined within a crater (~10^−17^ kg). At the same time, *E*_*J*_ linearly depends on the current flowing during the breakdown (~100–500 nA), breakdown voltage (~5–10 V) and τ_B_. Taking into account the above parameters, we find that τ_B_ should be of the order of 10^−6^–10^−5^ s. Such timescale is much shorter than the resolution of our setup, explaining why these local self-healing breakdowns do not produce spikes on voltage-current characteristics in Fig. 2. Second, we notice that there is a sharp tip visible in a center of almost each crater (see Fig. 7(b) and (c)). Third, we determine that the process of new crater emergence abruptly stops as soon as the jump in *I*-*V* curve happens. Such a behavior resembles the transition from an avalanche to a stable state in electrorheological fluid with carbon nanotubes exposed to a strong electric field^41, 42^. In those experiments a general rule was discovered, indicating that if the entropy production (or Joule heating) reaches its theoretically possible maximum then the system transits from an inherently unstable regime, in which the current paths emerge and get destroyed, to a stable regime, in which stable and highly conducting paths exist.

Taking into account these observations, we suggest the following explanation of the capacitor recovery process. As the applied voltage is ramped up the local self-healing breakdowns start to appear. They cause local destruction of alumina and aluminum electrodes and formation of sharp tips in the centers of the craters. These tips are residues of resistive links produced and subsequently burnt out in the craters due to the heat generated in them. At certain voltage the system reaches a situation when many conducting links occur. At this point the net resistance of the capacitor becomes low compared to the series resistor *R*_*0*_. Thus the voltage drop on the capacitor *V*_*s*_ gets reduced. Therefore, the current paths cannot be burned by Joule heating and a stable short of the capacitor is observed. Yet, it disappears if the voltage is reduced. This is a global self-healing effect that is our main discovery. It is interesting to note that the resistance of the nanocapacitors after the global breakdown is usually on the order of few MΩ, *i*.*e*. it is the same order of magnitude as *R*_*0*_ ≈ 10 MΩ. Hence, the amount of Joule heating generated in the capacitor stays close to the maximum^42^ *P*_*max*_ = *U* ^*2*^/*4R*_*0*_. Therefore, the state of the capacitor after the breakdown corresponds to the maximum entropy production principle^41, 43^.

In conclusion, we have studied the dielectric failure under high electric fields in Al/Al_2_O_3_/Al capacitors with alumina thickness equal to 4, 6.5 or 9 nm. Two types of the breakdown, namely a local and a global, have been observed. As a consequence of the local breakdown a nanometer-size defect appears on a surface of the top electrode. At the same time, it does not form a stable electrical connection between capacitor’s plates and does not cause any visible peculiarity on a voltage-current curve in our experiment. The reason is that a crater forms around the breakdown spot. The global breakdown manifests itself as a current jump on the current-voltage dependence. It leads to a formation of the short between the electrodes, which resistance typically adjusts such as to maximize the Joule heating, similarly to some previously studied self-organizing dissipative structures. We find that the studied nanocapacitors can recover after the global breakdown if the applied voltage is reduced. Such a recovery renders the capacitors with a few nanometer-thick insulating layer a promising candidate for energy storage applications.
